# A novel and efficient synthetic route to perfluoroisobutyronitrile from perfluoroisobutyryl acid†

**DOI:** 10.1039/c8ra07821a

**Published:** 2018-11-05

**Authors:** Yi Wang, Mengting Sun, Zhanyang Gao, Lilin Zou, Lingyu Zhong, Ruichao Peng, Ping Yu, Yunbai Luo

**Affiliations:** Engineering Research Center of Organosilicon Compounds & Materials, Ministry of Education, College of Chemistry and Molecular Sciences, Wuhan University Wuhan 430072 P. R. China ybai@whu.edu.cn

## Abstract

A novel synthetic route to perfluoroisobutyronitrile from perfluoroisobutyryl acid which has mild conditions and low toxicity is described. This study introduces detailed synthetic protocols and characterization including GC-MS, ^13^C NMR and ^19^F NMR spectroscopy of perfluoroisobutyryl acid, perfluoroisobutyryl chloride, perfluoroisobutyl amide and perfluoroisobutyronitrile. Besides, this route is superior to the established patent and shows potential application in high voltage electrical equipment.

## Abstract 2

The route of perfluoroisobutyronitrile from perfluoroisobutyryl acid and the route to raw materials.

## Graphical abstract

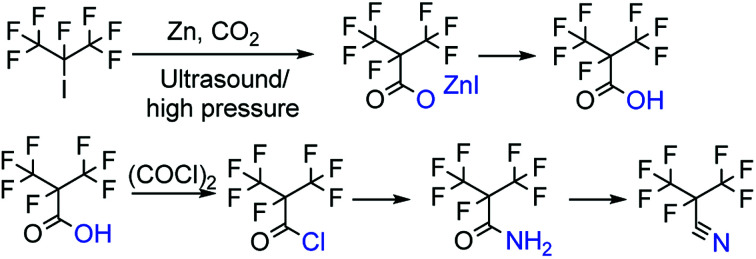

## Introduction

The gaseous compound sulfur hexafluoride (SF_6_) has a high dielectric strength (DS) and a good arc extinguishing performance. These features allow it to be widely used as a mainstream insulating gas which was previously used in GIS (Gas Insulated Switchgear), GIL (Gas Insulated Lines) or other large power equipment. However, it is also extremely harmful to the environment and the atmosphere because it takes a long time to decompose when it has been in the air. The GWP (Global Warming Potential) of SF_6_ is around 23 000 times that of CO_2_. It is expected to result in a stronger greenhouse effect.

The Kyoto Protocol, the supplement of the United Nations Framework Convention on Climate Change has declared SF_6_ as one of the six limiting gases. In order to overcome this issue, participating countries are urgently striving to find a new alternative compound with a low GWP as well as high DS that can completely replace SF_6_.^1–3^

On-going research in General Electric (GE), America has led to the emergence of some new insulating gases like CF_3_I, c-C_4_F_8_, i-C_5_F_10_O, and i-C_4_F_7_N. There are handful reports about the synthesis of i-C_4_F_7_N, furthermore most of them aren't practical to apply in industry.^4–11^ The gas i-C_4_F_7_N, when mixed with CO_2_, exhibits excellent properties, which surely be one of the first choices to replace SF_6_. Besides, the GWP of i-C_4_F_7_N is only 2210, which is far lower than that of SF_6_. Under the same pressure, insulation strength and arc extinguishing performance of i-C_4_F_7_N/CO_2_ can reach 90% or higher than that of SF_6_.

**Scheme 1:**
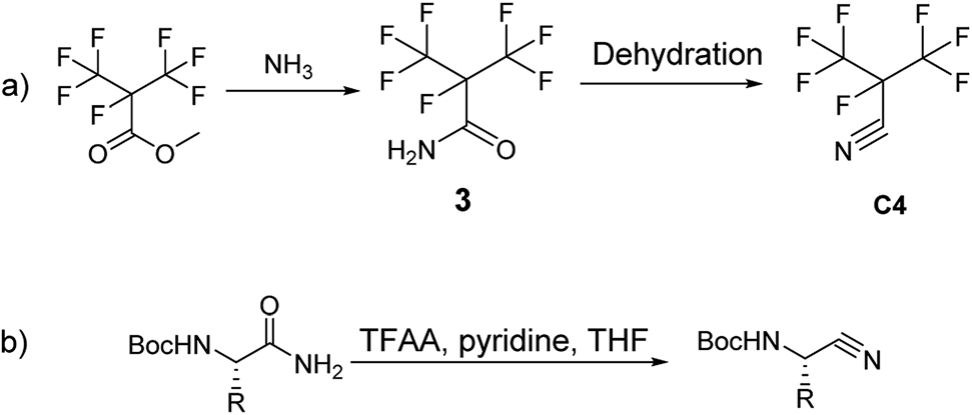
(a) The patent of 3 M; (b) synthesis of Boc-amino nitriles.

Recently, GE has built a 420 kV i-C_4_F_7_N/CO_2_ GIL. 3 M (Minnesota, Mining and Manufacturing) has issued a patent on the synthetic route to i-C_4_F_7_N (Scheme 1a).^12^ According to their protocol, methyl perfluoroisobutyrate was used as the initial starting material which was made to react with NH_3_ to form perfluoroisobutyl amide. The dehydration of the perfluoroisobutyl amide resulted in the formation of perfluoroisobutyronitrile. The dehydration of perfluoroisobutyl amide to produce its corresponding nitrile is an established synthetic strategy.

Sureshbabu had reported the general protocol for the synthesis of Boc-amino nitriles (Scheme 1b).^13^ The method was also found to be suitable for the synthesis of perfluoroisobutyronitrile.

Earlier attempts by Ishikawa to prepare organometallic compounds from perfluoroalkyl iodides and carbon dioxide yielded perfluoroalkyl acid as the organic product.^14^ Ishikawa had explained the mechanism for the formation of perfluoroalkyl acid and reported the ^19^F NMR of a series of perfluoroalkyl acids. However, the ^19^F NMR data of C_3_F_7_COOH had certain inaccuracies. Ishikawa had taken C_8_F_17_COOH as an example, and explained the detailed procedure of its preparation. Even so, the physical properties of C_3_F_7_COOH and C_8_F_17_COOH such as the boiling point or melting point are very different. In fact, it was difficult to obtain perfluoroisobutyryl acid following the general procedures given by Ishikawa.

To the best of our knowledge, only a handful of reports are available regarding the synthesis of perfluoroisobutyryl acid. One of the prime reasons behind this is the most of the researchers focused on the formation of perfluoroisopropylzinc iodide, but actually the concentration of CO_2_ also performed an important factor in the reaction. The scenario here is somewhat different from other organometallic reactions. We had even tried to purchase perfluoroisobutyryl acid from some commercial chemical companies, but interestingly, none of them were successful in synthesizing it, in spite of following the reported protocols. The other reason for this compound being so less studied is the fact that only a little was known about its practical value previously. However, considering Kyoto Protocol, perfluoroisobutyronitrile has a widespread application in GIL and other large-scale electrical equipment. Hence, it is highly likely for perfluoroisobutyryl acid to become a new raw material for the preparation of perfluoroisobutyronitrile.

So far, no study exists which reports the synthesis of perfluoroisobutyronitrile from perfluoroisobutyryl acid. Besides, there are no reports on the properties of perfluoroisobutyryl chloride. This study would be the first of its kind to report the details.

## Results and discussion

**Scheme 2:**
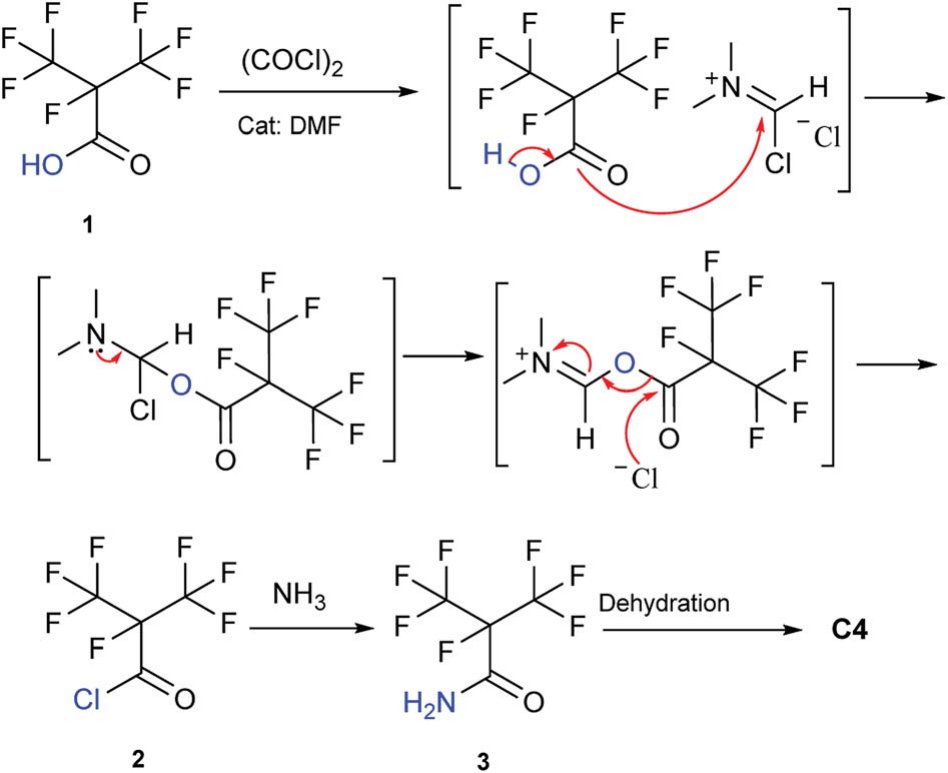
The synthetic route in this study.

**Fig. 1:**
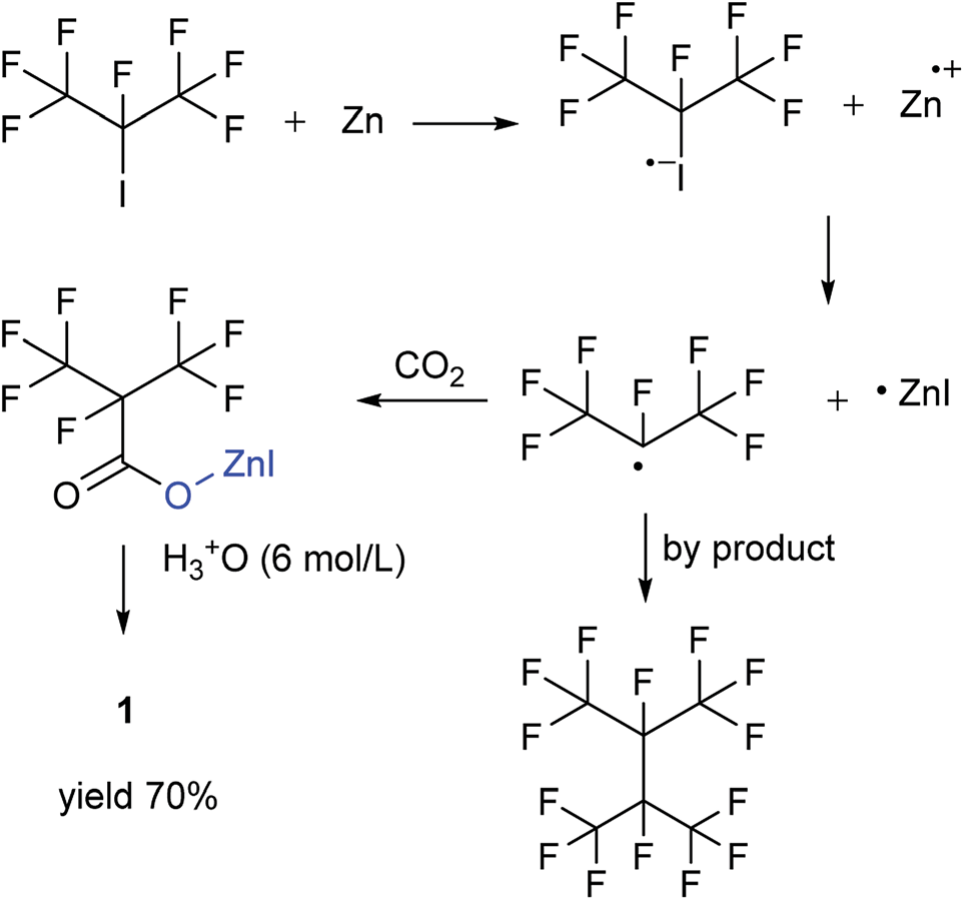
Synthesis of 1 from perfluoroisopropyl iodide.

Our attention was drawn to a novel synthetic route to i-C_4_F_7_N from a safer raw material where perfluoroisobutyryl acid was initially converted to the corresponding chloride which was a highly reactive intermediate (Scheme 2). The synthetic route to perfluoroisobutyryl acid (1) was shown in Fig. 1. In our work, the perfluoroisopropyl iodide was found to react readily with zinc powder and carbon dioxide in an aprotic solvent under an ultrasound condition. In additions the reaction could also take place under high pressure. In this study, we had used the two methods to prepare 1 (detail data and results of various conditions were shown in ESI†) and listed their conditions and yield.

For this reaction, carbon dioxide was found to react with zinc compounds only under ultrasound and high pressure which is 1 MPa or higher. Increasing the concentration of CO_2_ in the system determined the success of the step. Ultrasonic condition or high pressure has been proved to be indispensable, as the desired product could not be obtained under normal pressure without ultrasound or high pressure. Nevertheless, when the solution and dry ice were placed in a closed cylinder with a pressure of 1 MPa or higher, the formation of perfluorocarboxylic acid salt was observed. The summarized results were shown in Table 1.

**Table 1:** The results of various pressure to synthesize 1^a^

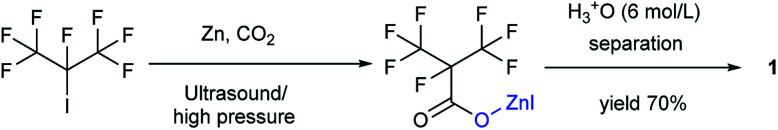		
Entry	Pressure^b^ (MPa)	Yield^c^ (%)
1	0	—d
2	1	24
3	2	33
4	3	69

a Reaction conditions: perfluoroisopropyl iodide (0.1 mol) in 30 mL DMF was added into 40 mL DMF including Zn powder at 50 °C for 2 h.

b Relative pressure in the reactor. The maximum pressure of reactor is 3 MPa.

c Isolated yield. Yields were determined by ^19^F NMR analysis using trifluorotoluene as an internal standard.

d No expected product was obtained.

Perfluoroisopropyl iodide was found to react readily with zinc powder in solution to form perfluoroisopropyzinc iodide, which further reacted with carbon dioxide under ultrasound or high pressure to give a final yield of nearly 70% 1. It was found that the synthesis at the reflux temperature was easier when the halide atom was iodide rather than bromide. On the other hand, the thermal stability of perfluoroisopropylzinc iodide in solution was in striking contrast to the instability by perfluoroisopropyzinc bromide.^15^

The amount of metal powder used in the reaction ranged from 1 to 4 equivalents of the raw material. It is evident that the yield of the reaction reached a plateau when the amount of the metal reached to more than 4 equivalents of the halogen in the raw material. Besides, the excessive metal powder in the solution rendered the separation step more difficult. In contrast, if the amount of the metal was less than 1 equivalent, the final solution was found to have a substantial amount of the starting material, since the metal powder was not enough to react with the entire reactant. In order to maximize the rate of conversion, and reduce the complexities during the separation, the most suitable amount should be 3 equivalents of the raw material. The zinc powder (diameter ranged from 1–3 mm) should be fully activated by 1 mol L^−1^ HCl (aq) and washed by acetone and ethanol. Then it was stored in the dry vacuum case until to be used.

Aprotic solvents such as *N*,*N*-dimethylformamide (DMF), tetrahydrofuran (THF), or diethyl ether used have been studied. DMF was found to be a convenient solvent because it could be hydrolyzed directly by water. Diethyl ether has an added advantage because its boiling point is lower than perfluoroisopropyl iodide.^16^ As a result, no remarkable amount of perfluoroisopropyl iodide was lost at the reflux temperature. Owing to the high saturation vapor pressure of diethyl ether, the combination of the solution and carbon dioxide became difficult. When using THF as the solvent, we found that perfluoroisopropyl iodide could not be dissolved in it. The purification of the solvent by molecular sieve was beneficial for the reaction. Direct use of anhydrous solvent was also a convenient method and gave satisfactory results.

The concentration of raw material in the solvent ranged from 1 mol L^−1^ to 3 mol L^−1^. The solvent-free method seems to be unsuitable because of the instability of perfluoroisopropyl iodide which evaporates quickly under ultrasound or high pressure due to the generation of heat. This would obviously result in the loss of a massive amount of raw material, thereby lowering the yield. If a very high quantity of the solvent is used, the concentration of raw material will become less than 1 mol L^−1^, which is not desirable. A high quantity of the solvent would also require a larger quantity of hydrochloric acid in the next step while lowering of the pH to enable the acidification of the perfluorocarboxylic acid salt.

We found that the most suitable reaction temperature was 60 °C. In order to prove the most suitable temperature, we carried out the reaction over a temperature gradient. The corresponding results are summarized in ESI.† The result illustrated that the reaction could not take place at low temperatures. The main impurity was found to be the raw material itself when the temperature was below 60 °C. Below 60 °C, most of the 1 will decompose to form hexafluoropropylene (HFP) in the distillation step.^17^ This would ultimately reduce the quantity of 1. In contrast, if the temperature exceeded 70 °C or higher, most of raw materials was lost during the reaction. Furthermore, the solution could not maintain the required concentration of carbon dioxide, as a result of which high conversion rates could not be obtained. In addition, the stability of the perfluorocarboxylic acid metal salt decreases at higher temperatures. Therefore, it was necessary to find an optimum temperature that could avoid thermal decarboxylation an effect that was accentuated by the decomposition to form HFP or dimers and trimers irreversibly.

Ultrasound has been already proven to be an essential requirement for the combination of carbon dioxide and metal compounds in promoting the reaction because ultrasound forms cavities that can readily dissolve carbon dioxide in solution. The pressure was also found to be an important factor in this study. It was found that the reaction could take place even without ultrasound, if the pressure was greater than 1 MPa. This observation could be justified on the basis of the fact that high pressures could reasonably increase the concentration of carbon dioxide in the solution. Either way, both the methods aimed at increasing the dissolution of the carbon dioxide in the solution.

The perfluoroisobutyrylzinc compound was so stable that it could be stored at room temperature with hardly any other reactions occurring. However, heating over 100 °C initiated its decomposition to form HFP. Hydrolysis of perfluoroisobutyrylzinc proceeded rapidly with the addition of water at room temperature. The products included Zn(OH)I and 1,1,1,2,3,3,3-heptafluoropropane (C_3_F_7_HF). C_3_F_7_HF was a gas phase at room temperature while the Zn(OH)I formed a precipitate in the sodium hydroxide solution. The precipitate of Zn(OH)I disappeared when then pH of the solution was made acidic. The reaction of perfluoroisobutyrylzinc compound with CO_2_ could take place under ultrasound or high pressure to form fluoroisobutyryl acid salt. The salt was converted into 1 after the addition of hydrochloric acid in order to adjust the pH to be less than 2. The final pure 1 was obtained after being separated, dried and distilled.

The choice of solvent in the step concerning the synthesis of 2 was versatile. The solvents used could be DMF or THF, and interestingly (Table 2), the reaction could also progress without the presence of any solvent. Different solvent showed different results. For example, DMF was found to catalyze this particular synthesis. Addition of high amounts of DMF made the reaction more vigorous. If THF was used as solvent, the vapour pressure of the solvent needed to be considered. This is because THF would be present even in the distillate, although it could be removed in next step of synthesizing the perfluoroisobutyl amide (3). The possible reason is that the boiling point of THF is lower than that of the amide. The absence of any solvent was acceptable. But storing the purified product would be difficult due to the vapour pressure and low boiling point.

**Table 2:** The result of various solvent to synthesize 2^a^

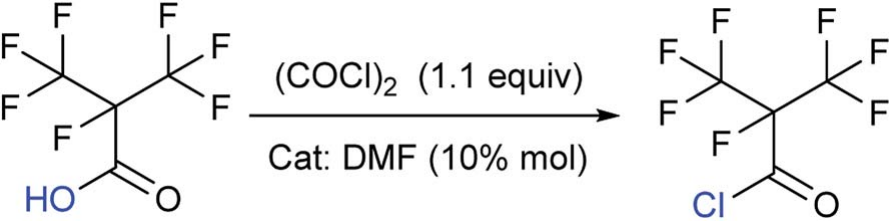		
Solvent	Purity^b^ (%)	Yield^c^ (%)
DCM	—	—d
DMF	52	6
THF	31	61
No solvent	99	60

a Reaction conditions: 1 (0.05 mol), oxalyl chloride (0.055 mol) in solvent (3 equiv) at 50 °C for 2 h.

b GC purity.

c Isolated yields.

d No expected product was obtained.

The synthesis of 2 didn't take place at low temperature. Contrarily, the high temperature was also not favorable for the reaction because of the low boiling point and high vapor pressure of 2. So the most suitable temperature was determined to be the initial reflux temperature.^18^ These properties also suggested that the storage of 2 was difficult. Therefore, a separation device was used to collect 2, which was made to drip into a methanol ammonia solution for the synthesis of 3. This apparatus allowed the reaction to proceed without any pause, which otherwise would have been essential if the advanced purification of 2 had to be done. This set up reduced the loss of perfluoroisobutyry chloride and improved the reaction efficiency. It therefore became convenient to purify the product. Once the 3 was prepared, the synthesis of perfluoroisobutryl amide and perfluoroisobutyronitrile was easy. Their synthesis could be proceeded using the routine procedures for amination and dehydration. The dehydration process of 4 in particular is very well established, and hence we have not included too many details here.

**Scheme 3:**
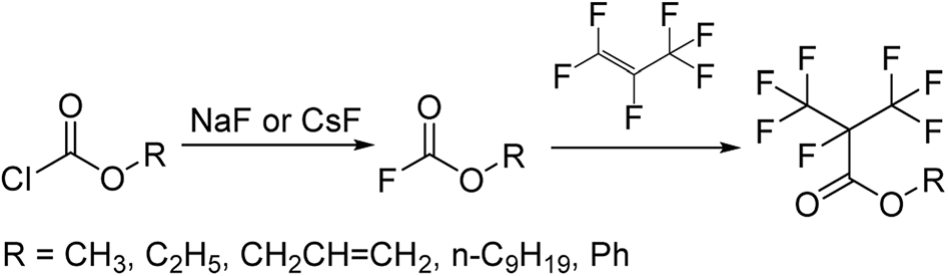
Synthesis of methyl perfluoroisobutyrate.^19^

Compared to this study, the Scheme 3 as the mainstream synthetic route of methyl perfluoroisobutyrate had many disadvantages and dangers. First, the raw material was chloroformate which was a highly toxic liquid that had been forbidden in many places. Second, the reaction in the gas–liquid phase in the next step wasn't easy to be controlled and operated. In fact, there was another way to synthesize C4. The electrolytic fluorination was also an effective route to prepare methyl perfluoroisobutyrate. But the route was more dangerous and had too many by-products to be separated. Our raw materials were easier to be purchased, stored and prepared. Furthermore, it had low chemical toxicity and good stability. Obviously, the methodology in Scheme 2 was simpler and safer to be used in the laboratory and its efficiency and yield were also no lower than any other methods.

## Experimental

### General remarks

Perfluoroalkyl halides were purchased from SHANG FLUORO. Zinc powder was purchased from Macklin Company and its diameter ranged from 1–3 m, purity was over 99.99%. Nearly 99.999% pure nitrogen gas and carbon dioxide gas were purchased from the WuHanShi XiangYun Industry Co. Ltd. All the solvents were of analytical grade and used without further purification, unless otherwise stated. Ultrasonication was performed on a GT SONIC-P3 ultrasonic apparatus. Y2UR-250 type high-pressure reactor used in this study was purchased from ShangHai YanZheng Instrument Co. Ltd.

NMR spectra of 1 and 2 were recorded on a Bruker Avance-III NMR spectrometer (^1^H: 400 MHz, ^19^F: 376 MHz, ^13^C: 100 MHz) with reference to an internal (^1^H, ^13^C: SiMe_4_) and an external standard (^19^F: CDCl_3_).

NMR spectra of 3 and C4 were recorded on at temperature 298 K on a Bruker Avance-III 500 MHz spectrometer equipped with a 5 mm BBFO probe, a ^19^F frequency of 470.59 MHz and a ^13^C frequency of 125.76 MHz. All ^19^F spectra were recorded with a recycle delay of 2 s, sweep width of 242 ppm, 220k acquisition data points, 4 scans. All ^13^C spectra were recorded with a recycle delay of 3 s, sweep width of 242 ppm, 32k acquisition data points, 256 scans. All NMR spectra were processed with 1 Hz line broadening.

GC-MS were carried out on Varian 450-GC Gas Chromatograph and Varian 320-MS TQ Mass Spectrometer. The Gas Chromatograph was equipped with a 30 m and 0.250 mm, 0.25 mm df, VF-5 column.

### Synthesis of perfluoroisopropyzinc iodide

19.5 g (0.3 mol, diameter 1–3 mm) zinc powder and 40 mL DMF was added to a round bottom flask containing a tube for introducing gas, a thermometer, a spherical condensing tube and a drop funnel. Nitrogen was introduced into the apparatus to prevent the system from air. 30 mL DMF solution containing 29.6 g (0.1 mol) perfluoroisopropyl iodide was added to the aforesaid mixture. Then the solution was heated at 60 °C for 2 h which included the feeding dropping time of about 1 h. To filter the metal powder, the perfluoroisopropylzinc iodide was obtained in DMF.

### Synthesis of perfluoroisobutyryl acid (1) by ultrasonic method

To a round bottom flask containing a tube for introducing carbon dioxide gas, a thermometer, a spherical condensing tube and a drop funnel, was added metal powder and 40 mL DMF. The gas-introducing tube was inserted into the solution to ensure a better contact between carbon dioxide and the solution. The ultrasound was kept on until the reaction was over. The speed of introducing carbon dioxide was about 0.1 L min^−1^. To the flask, 30 mL DMF solution containing 0.1 mol raw material was added into 19.5 g (0.3 mol, diameter 1–3 mm) zinc powder and 40 mL DMF. Then the solution was heated 60 °C for 2 h which included a dropping time of about 1 h. The solution was filtered to remove the remaining metal powder. After the reaction, 100 mL of 6 N hydrochloric acid was added for the hydrolysis, and pH was adjusted to be less than 2. The solution was now separated in two phases. After the lower organic layer was distilled, dried, filtered and collected, deep brown perfluoroisobutyryl acid was obtained.

### Synthesis of 1 by high-pressure method

19.5 g (0.3 mol, diameter 1–3 mm) zinc powder and 70 mL DMF was placed in a dry high-pressure closed cylinder. The cylinder needed to be equipped with a constant pressure drop funnel, and containing 30 mL DMF and 29.6 g (0.1 mol) perfluoroisopropyl iodide. Introduction of carbon dioxide maintained a high pressure from 1 MPa to 3 MPa in the cylinder. The later procedures and temperature were the same as the ultrasonic method.

Deep red liquid; ^13^C NMR (100 MHz, CDCl_3_): *δ* = 159.77, 159.57, 120.35, 120.07, 117.50, 117.23, 89.77, 89.44, 87.27. ^19^F NMR (376 MHz, CDCl_3_): *δ* = −75.47, −75.49, −181.38, −181.40, −181.42, −181.44, −181.46. MS (EI) *m*/*z*: 214 [M]^+^, 197 [M − OH]^+^, 150 [C_3_F_6_]^+^, 69 [CF_3_]^+^, 45 [COOH]^+^. IR (KBr): 1772.29 cm^−1^ (C=O).

### Synthesis of perfluoroisobutyryl chloride (2)

To take into consideration the properties of perfluoroisobutyry chloride, a water separation reflux device was used. 10.8 g (0.05 mol) perfluoroisobutyryl acid along with and 12.3 mL (0.15 mol, 3.0 equivalents) THF as solvent and 10% mol DMF as catalyst was added to a round bottom flask which included a thermometer, a drop funnel and downward distilling head with a serpentine reflux condenser. Notably, the cooling medium in the condenser was better below −10 °C. 5 mL (0.06 mol, 1.2 equivalents) of oxalyl chloride was dropped into the solution in 2 h at 40 °C. As the reaction progressed, a massive colorless perfluoroisobutyryl chloride was obtained below reflux condenser. The yield was nearly 60%.

Colorless liquid; ^13^C NMR (100 MHz, CDCl_3_): *δ* = 160.08, 159.88, 120.34, 120.08, 117.49, 117.23, 89.40, 87.23, 86.90. ^19^F NMR (376 MHz, CDCl_3_): *δ* = −75.94, −75.96, −75.98, −75.99, −168.99, −168.00, −168.01, −168.02, −168.03, −168.04, −168.05, −168.06, −168.07. MS (EI) *m*/*z*: 232 [M]^+^, 197 [M − Cl]^+^, 169 [C_3_F_7_]^+^, 100 [C_2_F_4_]^+^, 69 [CF_3_]^+^, 63 [COCl]^+^.

### Synthesis of perfluoroisobutyryl amide (3)

11.6 g (0.05 mol) perfluoroisobutyryl chloride solution was dropped into 22 mL 7.0 M (0.15 mol NH_3_ in solution) NH_3_·MeOH. The temperature was controlled below 20 °C. The reaction time was around 1 h. After the reaction, the main solid by-product was ammonium chloride, which was henceforth filtered. There was still a small amount of ammonium chloride dissolved in methanol. Methanol was removed by distillation. To add chloroform into solution at room temperature and filter all solid by-product. After being kept overnight in the refrigerator, perfluoroisobutyl amide crystals were found to precipitate out. The yield was 90%.

Colorless crystal; ^13^C NMR (126 MHz, CDCl_3_) *δ* = 159.69, 159.53, 120.05, 119.84, 117.76, 117.55, 89.50, 89.28, 87.80, 87.54, 87.24. ^19^F NMR (376 MHz, CDCl_3_): *δ* = −74.54, −74.55, −180.05, −180.06, −180.08, −180.09, −180.11, −180.12. MS (EI) *m*/*z*: 213 [M]^+^, 169 [C_3_F_7_]^+^, 69 [CF_3_]^+^, 44 [CONH_2_]^+^.

### Synthesis of perfluoroisobutyronitrile (C4)

10 g perfluoroisobutyryl amide and 12 mL pyridine was put in 20 mL DMF. Then 13.8 mL trifluoroacetic anhydride was dropped into the solution. The temperature was controlled below 0 °C. The reaction time was around 3 h. The 4.5 g perfluoroisobutyronitrile was obtained in ice trap. The yield was 49.5%.

Colorless gas; ^19^F NMR (376 MHz, CDCl_3_): *δ* = −75.37, −75.39, −176.49, −176.51, −176.53, −176.56, −176.58. MS (EI) *m*/*z*: 195 [M]^+^, 176 [C_4_F_6_CN]^+^, 107 [C_3_F_3_CN]^+^, 100 [C_2_F_4_]^+^, 57 [CFCN]^+^,69 [CF_3_]^+^, 31 [CF]^+^.

## Conclusions

In summary, we have developed a novel method for the synthesis of perfluoroisobutyronitrile from perfluoroisobutyryl acid which is a safer and more convenient route than established patent. The compound is introduced as the new environment-friendly insulating gas as the replacement of SF_6_ in high voltage electrical equipment, with the aim of abiding by Kyoto Protocol. Perfluoroisobutyryl acid could be synthesized with ultrasound and high pressure over 1 MPa at 60 °C. It reacted with (COCl)_2_ at 40 °C to form perfluoroisobutyryl chloride that then react with NH_3_ to get perfluoroisobutyryl amide. Raw materials and intermediates are characterized by GC-MS, ^13^C NMR ^19^F NMR spectra in the study.

## Conflicts of interest

There are no conflicts to declare.

## Supplementary Material

RA-008-C8RA07821A-s001
