# Supplementary material for: A novel and efficient synthetic route to perfluoroisobutyronitrile from perfluoroisobutyryl acid
Source: RSC Adv. 2018 Nov 5;8(65):37159–64. doi: 10.1039/c8ra07821a (PMC9088968; doi:10.1039/c8ra07821a)
Supplement: RA-008-C8RA07821A-s001 [file RA-008-C8RA07821A-s001.pdf]

Electronic Supplementary Material (ESI) for RSC Advances.
This journal is © The Royal Society of Chemistry 2018

Supplementary Information

A Novel and Efficient Synthetic Route to Perfluoroisobutyronitrile from Perfluoroisobutyryl Acid

Yi Wang, Mengting Sun, Zhanyang Gao, Lilin Zou, Lingyu Zhong, Ruichao Peng, Ping Yu and Yunbai Luo*

Engineering Research Center of Organosilicon Compounds & Materials, Ministry of Education, College of Chemistry and Molecular Sciences, Wuhan University, Wuhan, 430072, P. R. China.

E-mail: ybai@whu.edu.cn

1. **General materials** 2
2. **Synthesis of 1 (Scheme 3)** 3
3. **Synthesis of perfluoroisobutyryl chloride (2)** 6
4. **Synthesis of perfluoroisobutyryl amide (3)** 7
5. **Synthesis of perfluoroisobutyronitrile (C4)** 8

Appendix: ^{13}C, ^{19}F NMR and IR spectra of above compounds.

1. General materials

Perfluoroalkyl halides were purchased from SHANG FLUORO. Perfluoroalkyl halides were purchased from SHANG FLUORO. Zinc powder was purchased from Macklin Company and its diameter ranged from 1 ~ 3 m, purity was over 99.99%. Nearly 99.999 % pure nitrogen gas and carbon dioxide gas were purchased from the WuHanShi XiangYun Industry Co. Ltd. All the solvents were of analytical grade and used without further purification, unless otherwise stated. Ultrasonication was performed on a GT SONIC-P3 ultrasonic apparatus. Y2UR-250 type high-pressure reactor used in this study was purchased from ShangHai YanZheng Instrument Co. Ltd.

NMR spectra of **1** and **2** were recorded on a Bruker Avance-III NMR spectrometer (^{1}H: 400 MHz, ^{19}F: 376 MHz, ^{13}C: 100 MHz) with reference to an internal (^{1}H, ^{13}C: SiMe4) and an external standard (^{19}F: $CDCl_3$).

NMR spectra of **3** and **C4** were recorded on at temperature 298 K on a Bruker Avance-III 500 MHz spectrometer equipped with a 5 mm BBFO probe, a ^{19}F frequency of 470.59 MHz and a ^{13}C frequency of 125.76 MHz. All ^{19}F spectra were recorded with a recycle delay of 2 s, sweep width of 242 ppm, 220 k acquisition data points, 4 scans. All ^{13}C spectra were recorded with a recycle delay of 3 s, sweep width of 242 ppm, 32 k acquisition data points, 256 scans. All NMR spectra were processed with 1 Hz line broading.

GC-MS were carried out on Varian 450-GC Gas Chromatograph and Varian 320-MS TQ Mass Spectrometer. The Gas Chromatograph was equipped with a 30 m & 0.250 mm, 0.25 mm df, VF-5 column. The heat procedure was started from 60 ℃ to 200 ℃.

IR spectra were recorded on a NICOLET FTIR Spectrometer, 4000-400cm^{-1}.

2. Synthesis of 1 (Scheme 3)

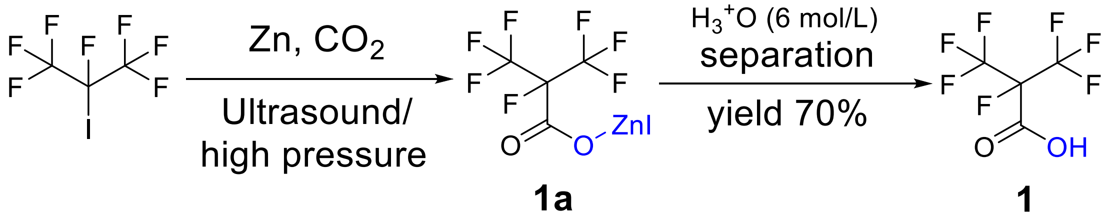

2.1 Synthesis of perfluoroisopropyzinc iodide (1a)

19.5 g (0.3 mol) zinc powder and 40 mL DMF was added to a round bottom flask containing a tube for introducing gas, a thermometer, a spherical condensing tube and a drop funnel. Nitrogen was introduced into the apparatus to prevent the system from air. 30 ml DMF solution containing 29.6 g (0.1 mol) perfluoroisopropyl iodide was added to the aforesaid mixture. Then the solution was heated for 2 h which included the feeding dropping time of about 1 h. To filter the metal powder, the perfluoroisopropylzinc iodide was obtained in DMF.

2.2 Synthesis of 1

2.2.1 Ultrasonic method:

To a round bottom flask containing a tube for introducing carbon dioxide gas, a thermometer, a spherical condensing tube and a drop funnel, was added metal powder and 40 ml DMF. The gas-introducing tube was inserted into the solution to ensure a better contact between carbon dioxide and the solution. The ultrasound was kept on until the reaction was over. The speed of introducing carbon dioxide was about 0.1 L/min. To the flask, 30 ml DMF solution containing 0.1 mol raw material was added. Then the solution was heated for 2 h which included a dropping time of about 1 h. The solution was filtered to remove the remaining metal powder. After the reaction, 100 ml of 6 N hydrochloric acid was added for the hydrolysis, and pH was adjusted to be less than 2. The solution was now separated in two phases. After the lower organic layer was distilled, dried, filtered and collected, deep brown perfluoroisobutyryl acid was

obtained. The experimental result is given in **Table 1**.

Table 1

Optimization of conditions for ultrasonic method to synthesize **1b**.[a]

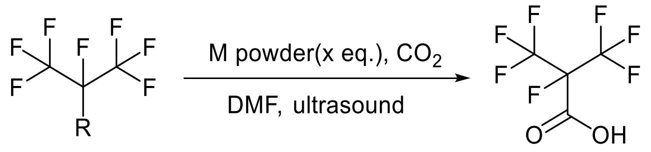

Entry	R	M	x	T (℃)	Power (W)	Yield (%)[b]
1	I	Zn	1	60	100	5
2	I	Zn	2	60	100	11
3	I	Zn	3	60	100	67
4	I	Zn	4	60	100	24
5	I	Zn	3	r.t.	100	-[c]
6	I	Zn	3	30	100	45
7	I	Zn	3	40	100	53
8	I	Zn	3	50	100	60
9	I	Zn	3	70	100	10
10	I	Zn	3	60	50	41
11	I	Zn	3	60	0	11
12	Br	Zn	3	60	100	5
13	I	Mg	3	60	100	-[d]
14	Br	Mg	3	60	100	-[d]

[a] Reaction conditions: 2.2 M (0.1 mol) perfluoroalkyl halide in 30 ml DMF was added into 40 ml DMF including metal powder under ultrasound for 2 h. The speed of introducing CO_2 was 0.1 L/min.

[b] Isolated yield. Yields were determined by ^{19}F NMR analysis using trifluorotoluene as an internal standard.

[c] No organic layer appeared.

[d] A complicated black mixture couldn't be separated.

2.2.2 High-pressure method:

0.3 mol metal powder and 70 ml DMF was placed in a dry high-pressure closed cylinder. The cylinder needed to be equipped with a constant pressure drop funnel, and containing 30 ml DMF and 29.6 g (0.1 mol) perfluoroisopropyl iodide. Introduction of carbon dioxide maintained a high pressure in the cylinder. The later procedures were

the same as the ultrasonic method. The experimental result is given in **Table 2**.

Table 2

Optimization of conditions for high-pressure method to synthesize **1b**. [a]

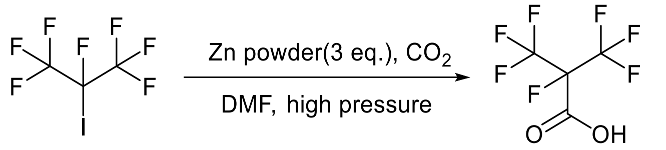

Entry	P (MPa) [b]	t (h)	T (℃)	Yield (%) [c]
1	1	1	50	24
2	2	1	50	33
3	3	1	50	46
4	3	2	50	69
5	3	3	50	50
6	3	2	60	49
7	3	2	70	39

[a] Reaction conditions: 2.2 M (0.1 mol) perfluoroisopropyl iodide in 30 ml DMF was added into 40 ml DMF including 19.5 g (0.3 mol) zinc powder under high pressure.

[b] Relative pressure in the reactor. The maximum pressure of reactor is 3 MPa.

[c] Isolated yield. Yields were determined by ^{19}F NMR analysis using trifluorotoluene as an internal standard.

Deep red liquid; ^{13}C NMR (100 MHz, $CDCl_3$): δ = 159.77, 159.57, 120.35, 120.07, 117.50, 117.23, 89.77, 89.44, 87.27. ^{19}F NMR (376 MHz, $CDCl_3$): δ = -75.47, -75.49, -181.38, -181.40, -181.42, -181.44, -181.46. MS (EI) m/z: 214 $[M]^+$, 197 $[M{-}OH]^+$, 150 $[C_3F_6]^+$, 69 $[CF_3]^+$, 45 $[COOH]^+$. IR (KBr): 1772.29 (C=O).

3. Synthesis of perfluoroisobutyryl chloride (2)

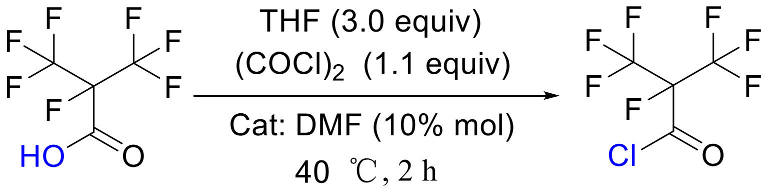

To take into consideration the properties of perfluoroisobutyry chloride, a water separation reflux device was used. 10.8 g (0.05 mol) perfluoroisobutyryl acid along with and 12.3 mL (0.15 mol, 3.0 equivalents) THF as solvent and 10% mol DMF as catalyst was added to a round bottom flask which included a thermometer, a drop funnel and downward distilling head with a serpentine reflux condenser. Notably, the cooling medium in the condenser was better below -10 ℃. 5 mL (0.06 mol, 1.2 equivalents) of oxalyl chloride was dropped into the solution in 2 h at 40 ℃. As the reaction progressed, a massive colorless perfluoroisobutyryl chloride was obtained below reflux condenser. The yield was nearly 60%.

Notification: $(COCl)_2$: b.p.: 62 - 65 ℃; m.p.: -10 - -8℃; 1.48 g/mL; Hazardclass: 8 --- 11; colorless liquid.

Colorless liquid; ^{13}C NMR (100 MHz, $CDCl_3$): δ = 160.08, 159.88, 120.34, 120.08, 117.49, 117.23, 89.40, 87.23, 86.90. ^{19}F NMR (376 MHz, $CDCl_3$): δ = -75.94, -75.96, -75.98, -75.99, -168.99, -168.00, -168.01, -168.02, -168.03, -168.04, -168.05, -168.06, -168.07. MS (EI) m/z: 232 $[M]^+$, 197 $[M-Cl]^+$, 169 $[C_3F_7]^+$, 100 $[C_2F_4]^+$, 69 $[CF_3]^+$, 63 $[COCl]^+$.

4. Synthesis of perfluoroisobutyryl amide (3)

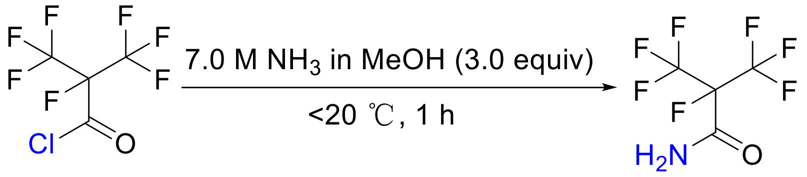

11.6 g (0.05 mol) perfluoroisobutyryl chloride solution was dropped into 22 mL 7.0 M (0.15 mol NH_3 in solution) NH_3•MeOH. The temperature was controlled below 20 ℃. The reaction time was around 1 h. After the reaction, the main solid by-product was ammonium chloride, which was henceforth filtered. There was still a small amount of ammonium chloride dissolved in methanol. Methanol was removed by distillation. To add chloroform into solution at room temperature and filter all solid by-product. After being kept overnight in the refrigerator, perfluoroisobutyl amide crystals were found to precipitate out. The yield was 90%.

Colorless crystal; ^{13}C NMR (126 MHz, $CDCl_3$) δ = 159.69, 159.53, 120.05, 119.84, 117.76, 117.55, 89.50, 89.28, 87.80, 87.54, 87.24. ^{19}F NMR (376 MHz, $CDCl_3$): δ = -74.54, -74.55, -180.05, -180.06, -180.08, -180.09, -180.11, -180.12. MS (EI) m/z: 213 $[M]^+$, 169 $[C_3F_7]^+$, 69 $[CF_3]^+$, 44 $[CONH_2]^+$.

5. Synthesis of perfluoroisobutyronitrile (C4)

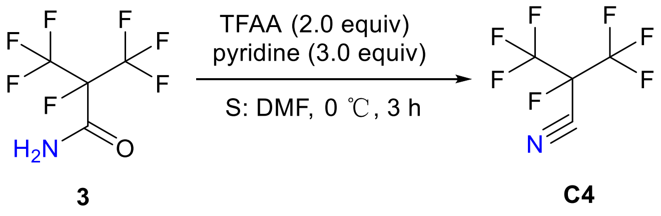

10 g perfluoroisobutyryl amide and 12 mL pyridine was put in 20 mL DMF. Then 13.8 mL trifluoroacetic anhydride was dropped into the solution. The temperature was controlled below 0 ℃. The reaction time was around 3 h. The 4.5 g perfluoroisobutyronitrile was obtained in ice trap. The yield was 49.5%.

Colorless gas; ^{19}F NMR (376 MHz, $CDCl_3$): δ = -75.37, -75.39, -176.49, -176.51, -176.53, -176.56, -176.58. MS (EI) m/z: 195 $[M]^+$, 176 $[C_4F_6CN]^+$, 107 $[C_3F_3CN]^+$, 100 $[C_2F_4]^+$, 57 $[CFCN]^+$,69 $[CF_3]^+$, 31 $[CF]^+$.

Appendix: ^{13}C and ^{19}F NMR spectra

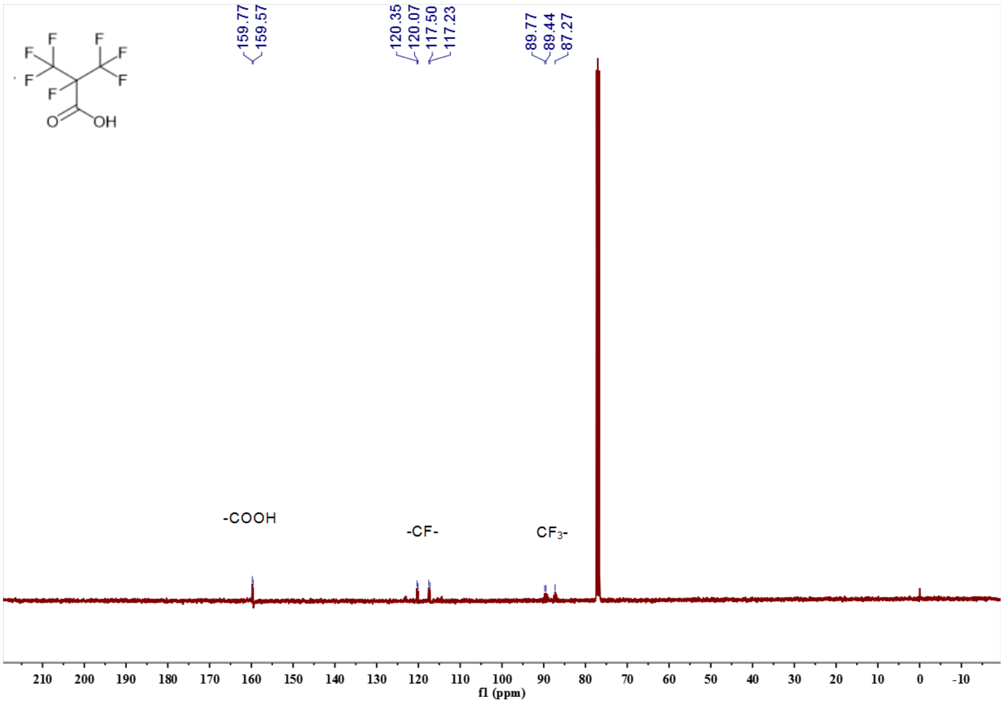

^{13}C NMR of **1** (100 MHz, $CDCl_3$)

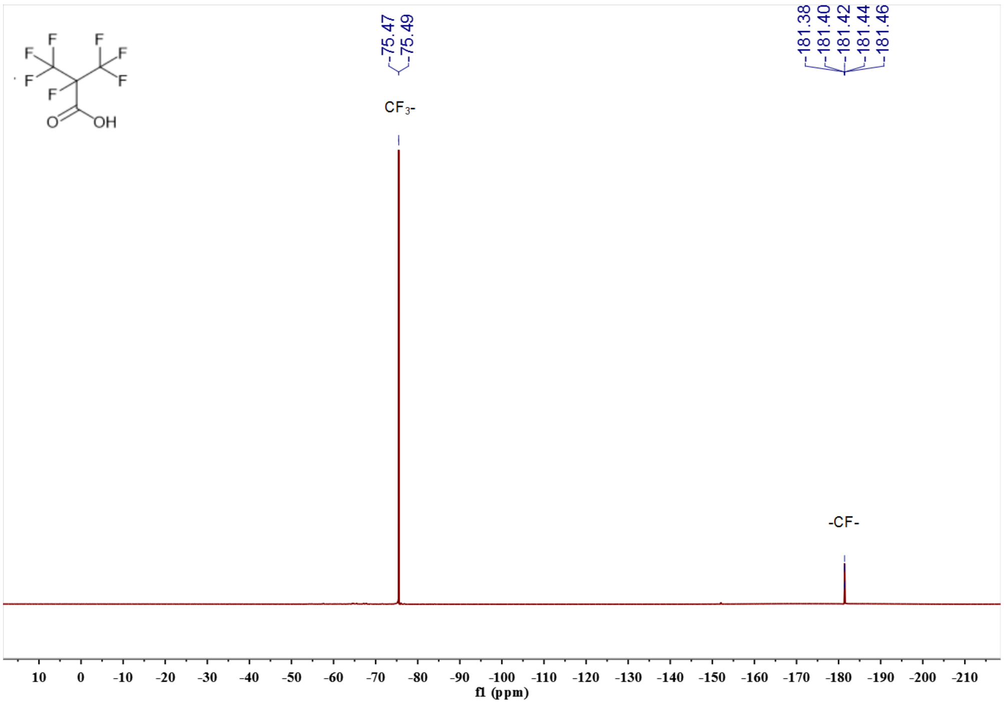

^{19}F NMR of **1** (376 MHz, $CDCl_3$)

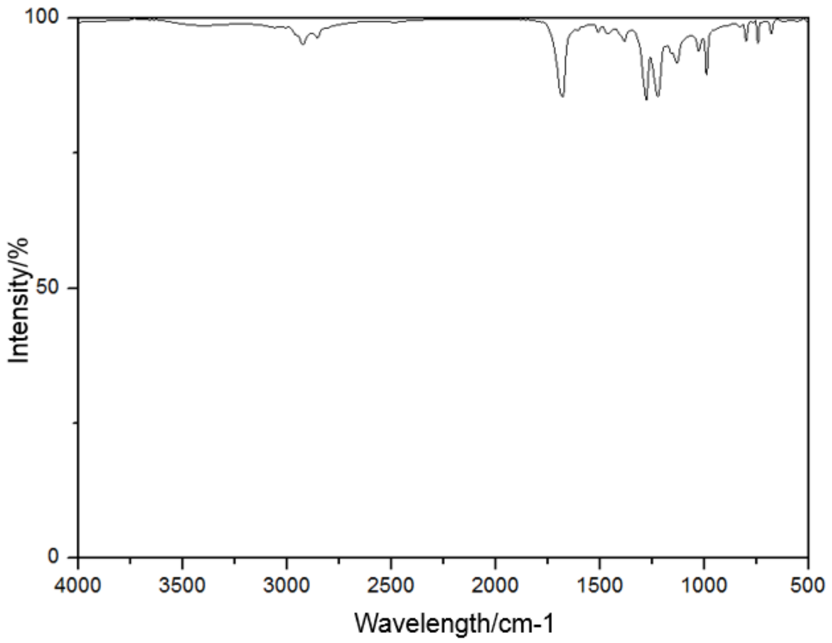

100
50
0
Intensity/%
4000
3500
3000
2500
2000
1500
1000
500
Wavelength/cm-1

IR of **1** (KBr)

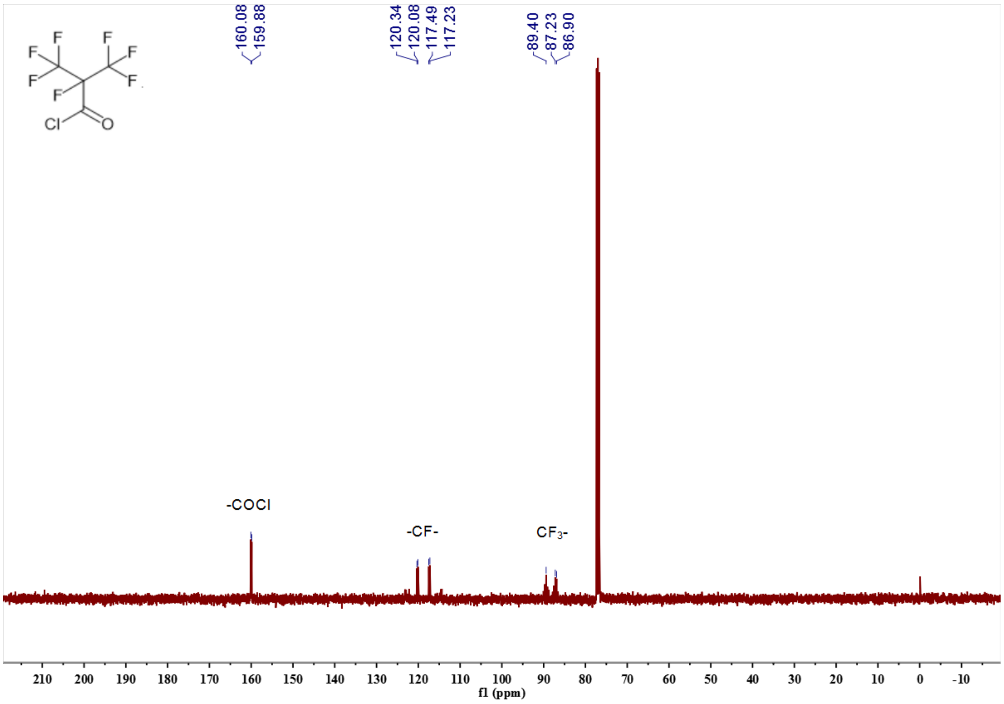

^{13}C NMR of **2** (100 MHz, $CDCl_3$)

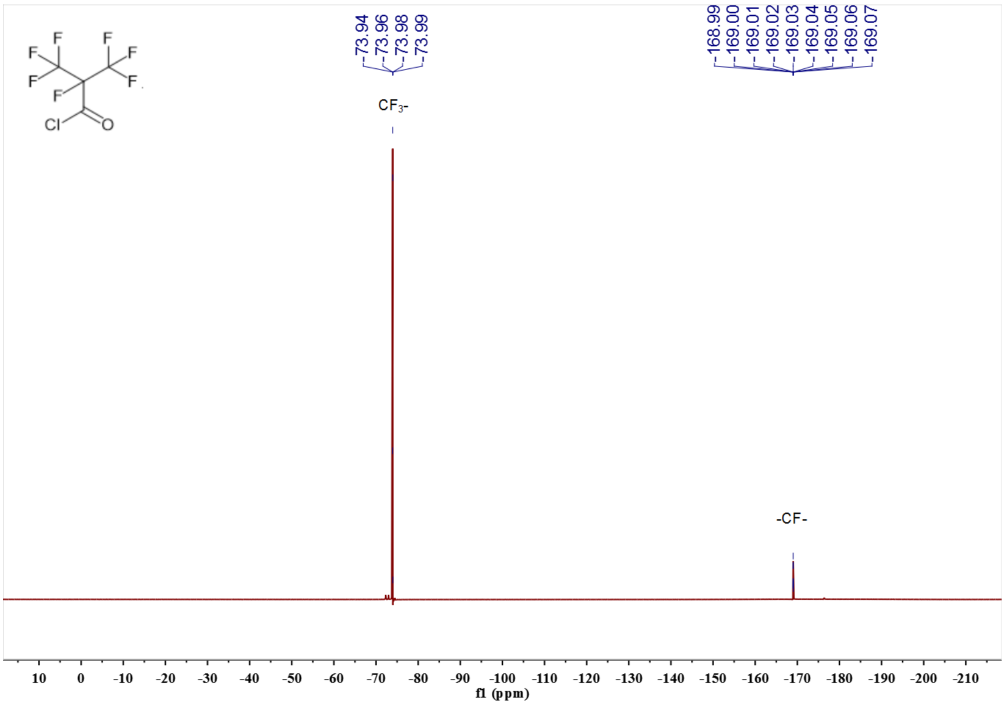

^{19}F NMR of **2** (376 MHz, $CDCl_3$)

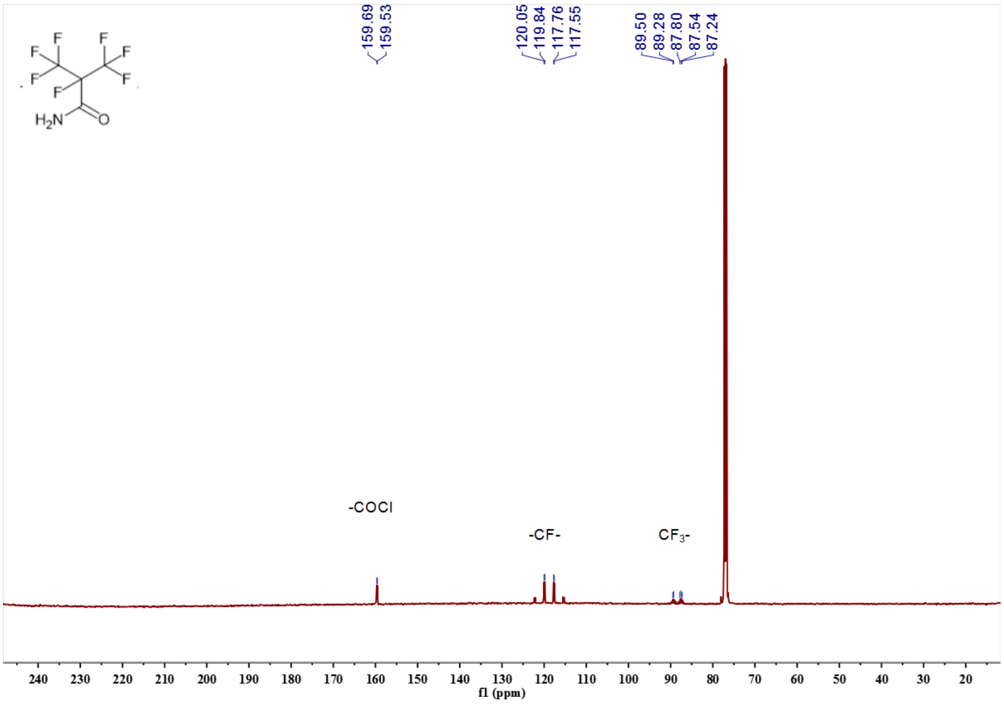

^{13}C NMR of **3** (125.76 MHz, $CDCl_3$)

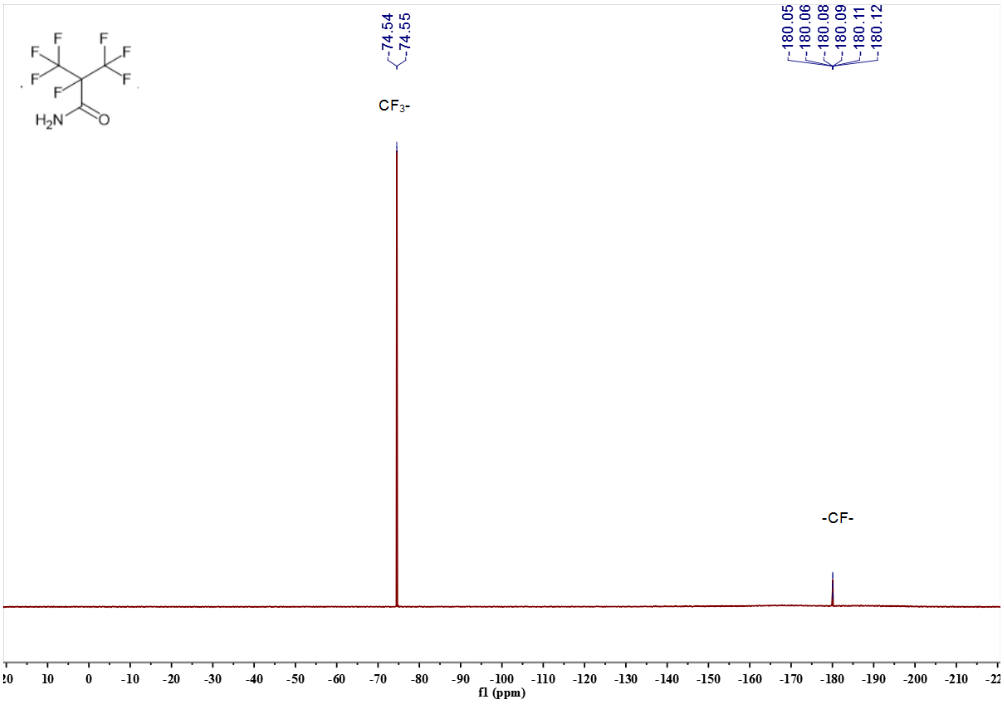

^{19}F NMR of **3** (470.59 MHz, $CDCl_3$)

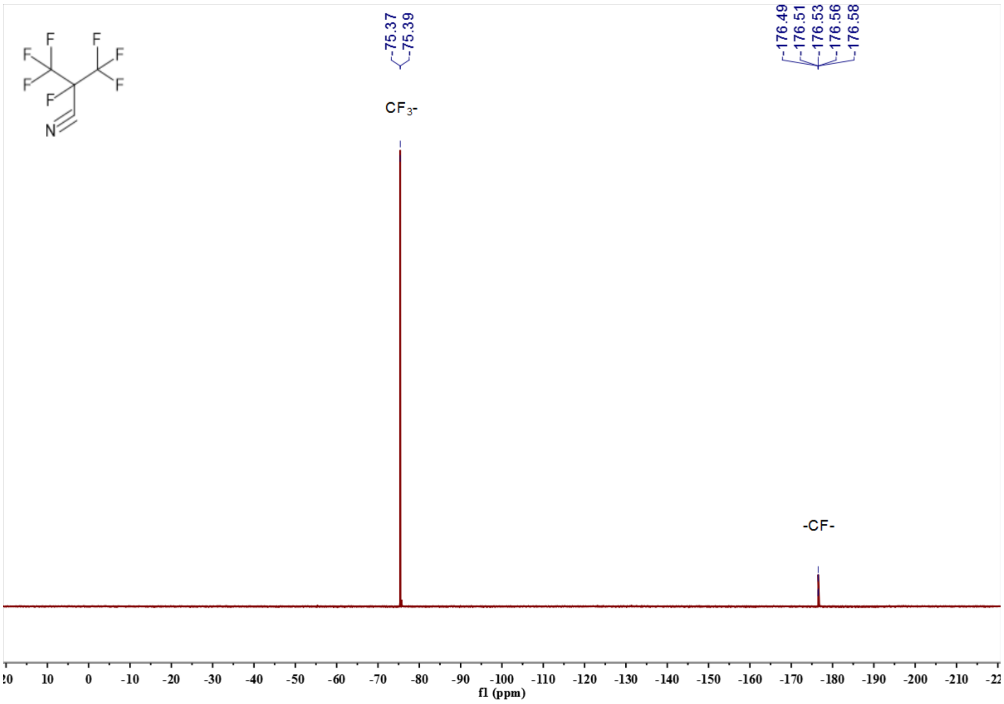

^{19}F NMR of **C4** (470.59 MHz, $CDCl_3$)
